# The incidence, risk factors, and outcomes of symptomatic avascular necrosis of bone among Chinese pediatric patients with acute lymphoblastic leukemia

**DOI:** 10.1002/cam4.5762

**Published:** 2023-03-31

**Authors:** Calvin Pui Lun Hoo, Alex Wing Kwan Leung, Joyce Pui Kwan Chan, Yin Ting Cheung, Frankie Wai Tsoi Cheng, Terry Tin Wai Chow, Grace Kee See Lam, Sau Yin Ha, Alan Kwok Shing Chiang, Rever Chak Ho Li, Chi Kong Li

**Affiliations:** ^1^ Department of Paediatrics and Adolescent Medicine Hong Kong Children's Hospital Hong Kong SAR China; ^2^ Department of Paediatrics, Faculty of Medicine The Chinese University of Hong Kong Hong Kong SAR China; ^3^ Hong Kong Hub of Paediatric Excellence The Chinese University of Hong Kong Hong Kong SAR China; ^4^ Department of Radiology Hong Kong Children's Hospital Hong Kong SAR China; ^5^ Department of Pharmacy, Faculty of Medicine The Chinese University of Hong Kong Hong Kong SAR China; ^6^ Department of Paediatrics and Adolescent Medicine The University of Hong Kong Hong Kong SAR China; ^7^ Department of Paediatrics and Adolescent Medicine Tuen Mun Hospital Hong Kong SAR China

**Keywords:** child, incidence, osteonecrosis, precursor cell lymphoblastic leukemia‐lymphoma, risk factors, young adult

## Abstract

**Background:**

Avascular necrosis (AVN) of bone is a debilitating complication of pediatric patients with acute lymphoblastic leukemia (ALL). While it is extensively studied and reported in Western population, studies focused on Orientals are limited. This study aims to evaluate the incidence, risk factors, and clinical outcomes of AVN among Chinese children with ALL.

**Methods:**

This study is a retrospective, territory‐wide population‐based cohort study of pediatric patients with ALL enrolled on one of the three consecutive ALL study protocols (ALL‐IC‐BFM 2002, CCLG‐ALL 2008, and CCCG‐ALL 2015).

**Results:**

A total of 24 out of 533 pediatric subjects with ALL (4.5%) had symptomatic AVN. Age was the single most important risk factor associated with the development of AVN. Only three patients were below age of 10 at the time of diagnosis of ALL. The incidences of AVN in patients aged above and below 10 years were 18.2% ± 3.6% and 0.8% ± 0.5%, respectively, and were significantly different (*p* < 0.005). Treatment protocol, immunophenotype, and gender were not predictive of AVN. Among the 24 patients, five required orthopedic interventions in view of progressive and severe disease. For subjects with hip joints involvement, follow‐up assessments showed 12 of 22 hip joints had radiological progression over a median duration of 3.63 years. Seventeen of them did not have pain at the latest follow‐up and among patients with pain (*n* = 7), five did not experience any limitation on activities of daily living while two required use of walking aids or wheelchair.

**Conclusion:**

The incidence of symptomatic AVN in Chinese ALL patients was comparable to other studies in Western population. Adolescent age more than 10 years old was recognized to be the most important factor for development of AVN. Significant proportion of patients had radiological progression over time with a small percentage of subjects had daily activities affected.

## Graphical abstract

This is a retrospective territory‐wide population‐based study on avascular necrosis in Chinese Pediatric patients in Hong Kong. The incidence is comparable with previous reports from Western countries and age is the most important risk factor.

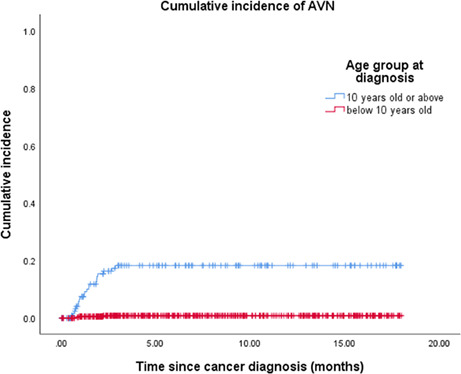

## 1 INTRODUCTION

Acute lymphoblastic leukemia (ALL) is the most common childhood cancer. Survival has dramatically improved over the last few decades. With contemporary treatment, the cure rate is now over 90%.^1^ Minimizing treatment‐related toxicities and morbidities is one of the main focuses in ALL management nowadays.^2^

Avascular necrosis (AVN) or osteonecrosis (ON) is one of the well‐known treatment‐related complications.^2^ The exact pathogenesis of AVN remains unclear, and postulated mechanisms include reduction in blood supply due to intramedullary fat overgrowth, micro‐emboli, and vasculitis.^3^, ^4^ AVN can result in long term pain and discomfort and adversely affect patients' quality of life.^5^ Surgical interventions and joint replacements may be necessary in severe cases.^6^

The reported incidence of AVN varied significantly among study groups and treatment protocols and ranged from 1.4% to 25% for symptomatic cases.^2^, ^3^, ^6^, ^7^, ^8^, ^9^, ^10^, ^11^, ^12^ The difference in incidence could be accountable by the difference in study population, treatment factors, timing of radiological investigation, and imaging modality used to diagnose AVN.^11^ It was reported that the risk of AVN differed between ethnic groups, and was more common in White than in Blacks.^3^, ^13^, ^14^ There were few studies investigating this complication in Orientals. A survey in UK suggested Asian had the highest risk of ON, whereas the Japan Association of Childhood Leukemia Study (JACLS) group reported a low incidence rate of 1.5% among patients treated with ALL‐97 and ALL‐02 protocols. But subsequent JACLS ALL‐08 protocol based on Berlin‐Frankfurt‐Munster (BFM) 95 protocol showed a cumulative incidence comparable to Western groups.^2^, ^15^, ^16^, ^17^ In the recent report from Chinese Children's Cancer Group (CCCG) on CCCG‐ALL‐2015 study on 6141 patients of Chinese ethnic background, 0.1% and 0.6%–0.8% subjects had grade 3–4 symptomatic AVN in the low risk and intermediate‐to‐high risk treatment group, respectively.^12^ The incidence of AVN in Chinese children with ALL appeared to be lower than that in Western populations.

The reported risk factors for development of AVN vary substantially among different studies.^7^ The most consistent and significant risk factor is age at diagnosis, with age greater than 10 years at increased risk.^7^, ^9^, ^11^, ^18^, ^19^ Cumulative dose and schedule of administration of corticosteroids are associated with development of AVN.^6^, ^7^, ^13^, ^20^ Association with other factors including sex, body mass index, and use of high dose methotrexate and asparaginase were reported but the findings were not consistent across studies.^6^, ^8^, ^14^, ^21^, ^22^

Magnetic resonance imaging (MRI) is now regarded as the standard method for diagnosing AVN.^6^, ^7^, ^14^, ^22^, ^23^ In patients with imaging features of AVN, it is recognized that some changes may remain stable over time, while some will progress.^11^ Similarly, clinical outcomes of patients with AVN varied significantly, ranged from transient and self‐limiting symptoms to debilitating joint damage requiring surgical intervention.^14^ It is challenging to identify patients at risk of functional impairment and progressive joint disease.

This study aims at identifying the incidence, risk factors, and clinical outcomes of AVN among Chinese pediatric patients with ALL in Hong Kong.

## 2 METHODS

This is a population‐based territory‐wide retrospective cohort study. Our study included Chinese pediatric patients aged less than 19 years at the time of ALL diagnosis. We recruited patients with ALL diagnosed before December 31, 2019 in order to ensure sufficient follow‐up period. Exclusion criteria were non‐ALL and non‐Chinese patients. The clinical data was censored on December 31, 2020.

Subjects were identified from three ALL study trials' databases including ALL‐IC‐BFM 2002,^24^ CCLG‐ALL 2008,^25^ and CCCG‐ALL 2015^12^ which were conducted between 2002–2008, 2009–2015, and 2015–2020, respectively. Dosage of steroid and asparaginase in these protocols are summarized in Table 1. Patients were followed up with collection of data on significant complications including AVN. In ALL‐IC‐BFM 2002 and CCCG‐ALL‐2015 study, AVN was a reportable adverse event. Data were cross‐checked at the Hong Kong Hospital Authority's electronic patient record (EPR) and clinical data analysis and reporting system. Only symptomatic patients were included in the study. There was no radiological screening for AVN in asymptomatic patients. Patients with AVN diagnosed after hematopoietic stem cell transplantation (HSCT) were excluded from analysis.

**TABLE 1:** Protocol specified dose of steroid and l‐asparaginase across IC BFM 2002, CCLG ALL 2008, and CCCG ALL 2015 studies.

	l‐Asparaginase dose (unit/m^2^)	Cumulative steroid dose equivalent^a^ (mg/m^2^)	Prednisolone (mg/m^2^)	Dexamethasone (mg/m^2^)
IC‐BFM 2002 protocol				
SR1	80,000	3411	1838	236
SR2	120,000	4052	1838	333
IR1	80,000	3411	1838	236
IR2	160,000	5159	1838	499
HR1	310,000	7157	1838	799
HR2A	270,000	8306	1838	971
CCLG ALL 2008 before 1‐1‐2014				
LR	80,000	5082	420	700
IR	120,000	M: 6294; F: 4522	420	M: 882; F: 616
HR	380,000	M: 8278; F: 7679	420	M: 1180; F: 1090
CCLG ALL 2008 after 1‐1‐2014				
LR	80,000	6281	420	880
IR	120,000	M: 6680; F: 5482	420	M: 940; F: 760
HR	380,000	M: 8278; F: 7679	420	M: 1180; F: 1090
CCCG ALL 2015				
LR	180,000	Arm A: 7793 Arm B: 5556	1080	Arm A: 1008 Arm B: 672
IR/HR	432,000	Arm A: 7887 Arm B: 5689	1080	Arm A: 1022 Arm B: 692
T‐cell ALL or Day 19 MRD ≥ 1%	468,000	Arm A: 8247 Arm B: 6049	1440	Arm A: 1022 Arm B: 692

Abbreviations: ALL, acute lymphoblastic leukemia; CCCG, Chinese Children's Cancer Group; HR, high risk; IR, intermediate risk; LR, low risk; SR, standard risk; M, male; F, female; MRD, minimal residual disease.

^a^ Steroid dose equivalent is calculated with the assumption of 1 mg dexamethasone is equivalent to 6.67 mg of prednisolone. CCLG ALL 2008 had protocol amendment on 1‐1‐2014 with all patients in IR and HR received pulse dexamethasone in maintenance therapy.

Patient demographics and treatment factors including age, sex, immunophenotype, treatment protocol, National Cancer Institute (NCI) risk group, and cumulative doses of corticosteroid and asparaginase at the time of AVN diagnosis were collected. Cumulative steroid exposure was calculated based on the assumption of 1 mg dexamethasone equivalent to 6.67 mg prednisolone.^18^ Patients were classified into AVN and non‐AVN groups for comparison.

Symptoms including joint pain, limping, and reduction in joint motion would raise clinical suspicion of AVN and MRI of the affected sites would be performed. The date of first reported MRI with features compatible with AVN was regarded as the date of AVN diagnosis. A single radiologist centrally reviewed all the X‐rays and MRIs at diagnosis and follow‐up and graded the severity according to Association Research Circulation Osseous Classification (ARCO) system.^26^ Patient's medical background and treatment‐related information were blinded to the radiologist.

Clinical outcomes parameters including ARCO staging, management (conservative or surgical interventions), pain, and activities of daily living were collected and analyzed. The information was retrieved from EPR or during follow‐up.

This study was approved by the Research Ethics Committee (REC) in Hong Kong Children's Hospital (REC reference number: HKCH‐REC‐2020‐025). Waive of consent for this retrospective study was approved by the REC as some subjects had passed away or lost to follow and were not available for consent while informed consents were obtained for all subjects upon enrollment on IC‐BFM‐2002, CCCG‐ALL‐2008, or CCLG‐ALL‐2015 study.

### 2.1 Statistical analysis

Continuous variables were expressed as means and standard error. For comparison between AVN group and non‐AVN group, the Chi‐square test or Fisher's exact test was used for categorical variables, and the Mann–Whitney *U* test for continuous variables. The cumulative incidence of AVN for each treatment protocol and age group at diagnosis (≥10 years vs. <10 years) was estimated using the Kaplan–Meier analysis. Events including AVN, death, and HSCT were censored. Among the patients with AVN, an exploratory analysis was conducted to identify risk factors associated with worse outcomes (progressive disease vs. improvement/ stable disease). A *p*‐value < 0.05 would be considered significant for all comparisons. Data were analyzed with SPSS version 25 (IBM).

## 3 RESULTS

A total of 553 patients were diagnosed with ALL in the period from January 1, 2003 till December 31, 2019, 20 subjects were excluded for non‐Chinese ethnicity. Among the remaining 533 subjects (305 males and 228 females), 169, 214, and 150 patients were enrolled on to ALL‐IC‐BFM 2002, CCLG‐ALL 2008, and CCCG‐ALL 2015 study, respectively. Details of the study protocols and treatment outcomes were described elsewhere.^12^, ^24^, ^25^

### 3.1 Incidence

**FIGURE 1:**
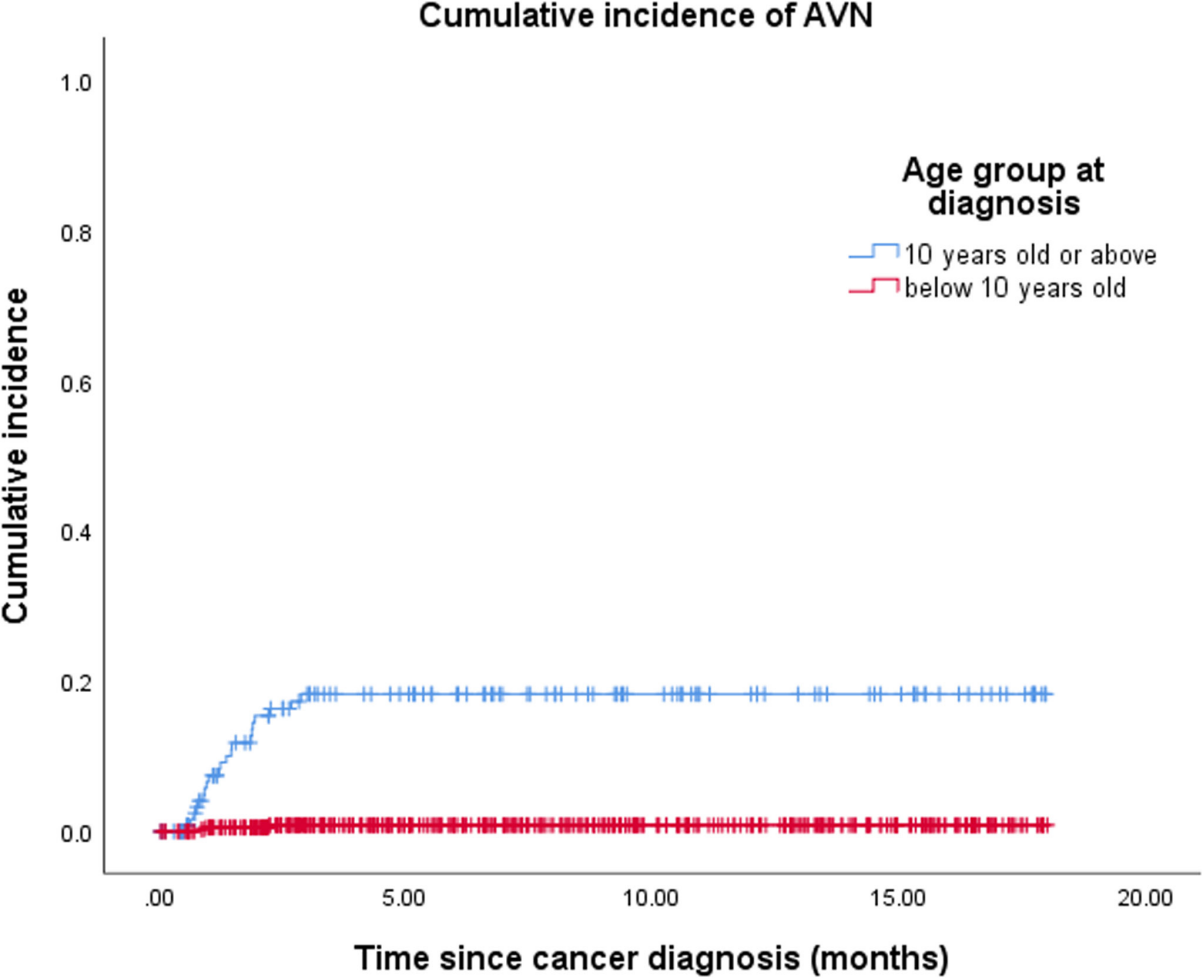
Cumulative incidence of avascular necrosis. Five‐year cumulative incidence for age ≥10 years and <10 years old was 18.2% ± 3.6% and 0.8% ± 0.5%, respectively.

**FIGURE 2:**
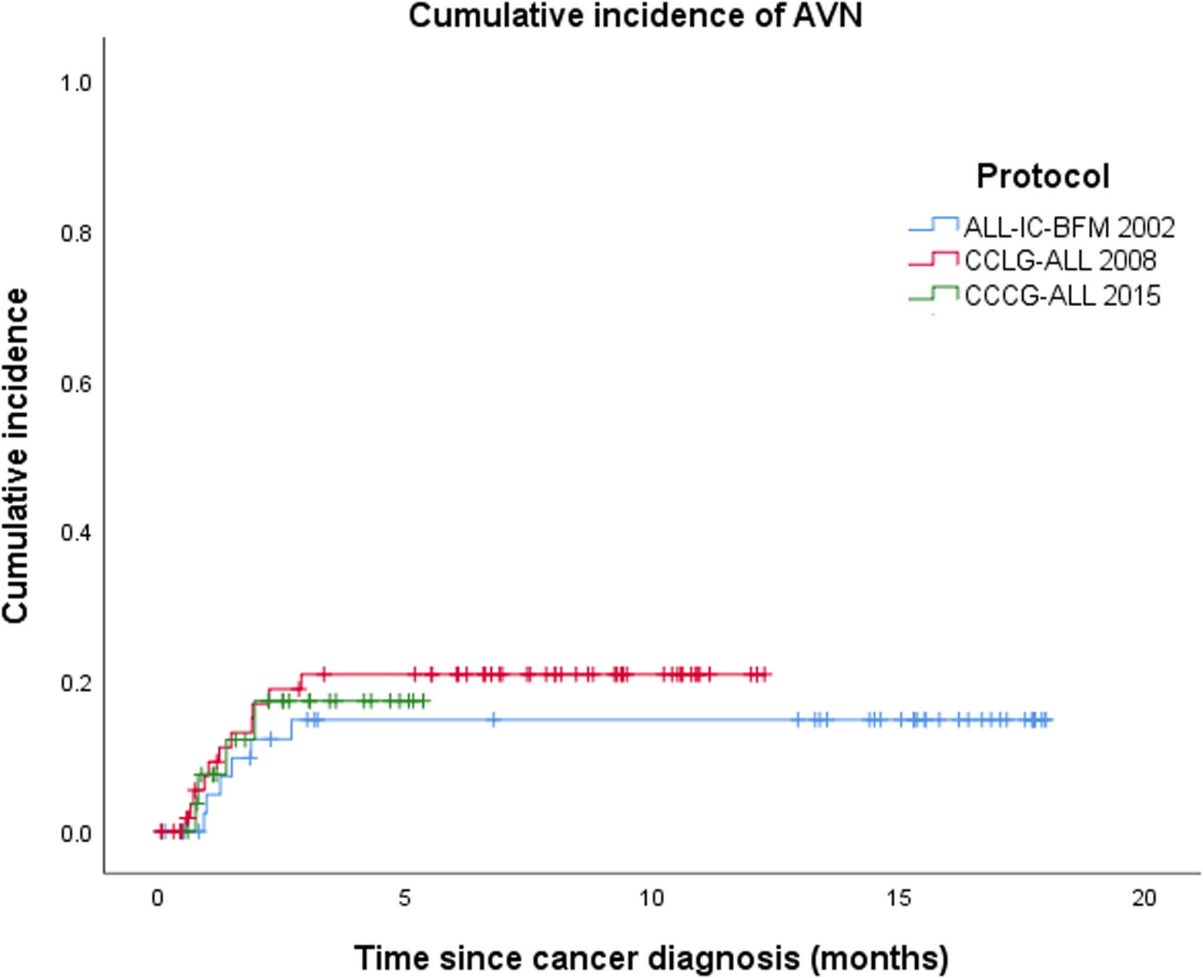
Avascular necrosis in age group ≥10 years. Five‐year cumulative incidence were 14.8%, 20.9%, and 17.3% for ALL‐IC‐BFM 2002, CCLG‐ALL 2008, and CCCG‐ALL 2015 protocols, respectively. CCCG, Chinese Children's Cancer Group.

Twenty‐four patients (4.5%) were diagnosed with symptomatic AVN. Two patients had AVN diagnosed after HSCT were not included in the analysis. Thirteen (54%) were male. Among 395 patients age <10 years, only three developed AVN, whereas 21 out of 138 subjects age ≥10 years were diagnosed to have AVN. Demographics and clinical characteristics for patients with AVN and without AVN are listed in Table 2. This constituted a 5‐year cumulative incidence of AVN of 0.8% ± 0.5% in subjects <10 years old and 18.2% ± 3.6% in ≥10 years old (Figure 1). The mean age at time of diagnosis of ALL was 14.7 ± 3.4 years in the AVN group and was significantly higher as compared with that of non‐AVN group (6.6 ± 4.5 years) (*p*‐value < 0.001). The cumulative incidence of AVN ≥ 10 years for the ALL‐IC‐BFM 2002, CCLG‐ALL 2008, and CCCG‐ALL 2015 protocols were 14.8%, 20.9%, and 17.3%, respectively and not significantly different (Figure 2).

**TABLE 2:** Demographics and clinical characteristic for patients with and without AVN.

	Patients with AVN, *N* = 24	Patients without AVN, *N* = 509	*p*‐value
	*n* (%)	*n* (%)	
Age at ALL diagnosis (mean ± SE)	14.7 ± 3.4 years	6.6 ± 4.5 years	<0.001
≥10 years	21 (87.5%)	117 (23%)	
<10 years	3 (12.5%)	392 (77%)	
Gender			0.757
Male	13 (54.2%)	292 (57.4%)	
Female	11 (45.8%)	217 (42.6%)	
Immunophenotype			0.007
B‐cell	17 (70.8%)	452 (88.8%)	
T‐cell	7 (29.2%)	56 (11%)	
NCI risk group			<0.001
Standard risk	2 (8.3%)	320 (62.9%)	
High risk	22 (91.7%)	189 (37.1%)	
Treatment protocol			0.356
IC BFM 2002	6 (25%)	163 (32%)	
CCLG ALL 2008	13 (54.2%)	201 (39.5%)	
CCCG ALL 2015	5 (20.8%)	145 (28.5%)	
Phase of treatment at AVN diagnosis			
Consolidation	1 (4.2%)		
Re‐induction	4 (16.7%)		
Maintenance	17 (70.8%)		
Post‐treatment (all within 1 year)	2 (8.3%)		

Patients age ≥10 years	With AVN	Without AVN	
	*N* = 21	*N* = 117	
Gender			0.523
Male	11 (52.4%)	70 (59.8%)	
Female	10 (47.6%)	47 (40.2%)	
Treatment protocol			0.741
ALL‐IC‐BFM 2002	6 (28.6%)	42 (35.9%)	
CCLG‐ALL 2008	11 (52.4%)	51 (43.6%)	
CCCG‐ALL 2015	4 (19%)	24 (20.5%)	
Immunophenotype			0.307
B cell	15 (71.4%)	87 (74.4%)	
T cell	6 (28.6%)	30 (25.6%)	
Cumulative dexamethasone dose at AVN diagnosis mean ± SE (mg/m^2^)	570.7 ± 355.6	NA	NA
Cumulative steroid equivalent dose at AVN diagnosis mean ± SE (mg/m^2^)	4430.1 ± 1977.4	NA	NA
Cumulative asparaginase dose at AVN diagnosis mean ± SE (units/m^2^)	212,636.3 ± 135,372.6	NA	NA

Abbreviations: ALL, acute lymphoblastic leukemia; AVN, avascular necrosis; CCCG, Chinese Children's Cancer Group; NA, not applicable; NCI, National Cancer Institute; SE, standard error.

### 3.2 Diagnosis

For the timing of AVN diagnosis, 22 of 24 patients developed AVN while still receiving chemotherapy. Five patients developed AVN during consolidation and re‐induction, which were during the first 6–8 months of treatment. The other 17 patients had AVN diagnosed at the later maintenance phase of treatment. The remaining two patients developed AVN within 1 year after completion of therapy. The median time from diagnosis of ALL to that of AVN was 1.24 years (range, 0.54–2.89 years). The mean steroid (expressed as prednisolone equivalent) and l‐asparagainase dose up to the time of AVN diagnosis were 4430 mg ± 1977 mg and 212,636 units ± 135,372 units, respectively. (Table 2).

### 3.3 Risk factors

Age at diagnosis was significantly association with the occurrence of AVN. As majority of AVN patients were over 10 years of age, the analyses were limited to this subgroup to minimize the dilution effects of younger children. Patient's demographics and treatment factors were summarized in Table 2. There was no significant difference in rate of AVN between patients with T‐ALL and B‐ALL (16.6% vs. 14.7%; *p* = 0.307). Other factors including sex (*p* = 0.523) and treatment protocols (*p* = 0.741) did not show any statistically significant difference between groups with and without AVN.

### 3.4 Imaging

All of the 24 patients suffered from multiple joints involvement. Notably hip, knee, and shoulder joints were the most frequently affected, with 22 hip joints in 12 patients, 27 knee joints in 15 patients, and 5 humeral head in 3 patients. Other affected sites include medullary infarct of femur and tibia (*n* = 17), pelvic bone (*n* = 3), ankle joints (*n* = 1), and vertebral bodies (*n* = 1).

Hip joint was the only site that had standardized classification systems to describe the severity of involvement.^26^ At diagnosis, about half (12 out of 22 joints) were at ARCO stage 1 and 2, while nine joints were having stage 3 disease. There was no stage 4 disease at the time of diagnosis.

### 3.5 Treatment and outcomes

All of the recruited subjects were followed up after completion of chemotherapy. The median follow‐up duration was 6.5 years (range, 1.1–13.6 years).

Patient's treatment, clinical and radiological outcomes were summarized in Table 3. Eighteen patients were managed conservatively with bed rest, “off‐loading” as tolerated, and analgesic for pain control. In patients still on‐therapy, 11 had modifications of treatment by either substituting prednisolone for dexamethasone (*n* = 2) or stopping the use of steroid in the remaining chemotherapy (*n* = 9). For radiological outcome, the latest ARCO staging was evaluated for all patients with hip involvement (*n* = 12) and the findings were compared with that at diagnosis. (Table 4) Seven out of twelve patients with hip joint involvement had radiological deterioration with 14 of 22 hip joints (63.6%) getting worse over a median period of 3.6 years (range, 0.5–9.4 years). Seven (31.8%) hip joints progressed to stage 4. Only two (9%) showed improvement and six (27.2%) remained static on follow‐up.

**TABLE 3:** Intervention, symptom, functional, and radiological outcome of patients with AVN.

	Total number of patients: 24, *n* (%)	
Intervention		
Conservative	18 (75%)	
Modification or stopping of steroid in remaining chemotherapy treatment	11 (45.8%)	
Operation		
Core decompression	6 (25%)	
Arthroplasty	1 (4.1%)	
Long term pain		
Yes	7 (29.1%)	
No	17 (70.8%)	
Activities of daily living		
Independent and no limitation	22 (91.7%)	
Independent with some limitation in mobility requiring walking aid/wheelchair	2 (8.3%)	
Dependent	0 (0%)	
Radiological outcome	22 Hip joints, *n* (%)	19 Joints/sites other than hip joints, *n* (%)
Improvement	2 (9.1%)	7 (36.8%)
Static	6 (22.7%)	4 (21.1%)
Progression	14 (59.1%)	8 (42.1%)

Abbreviation: AVN, avascular necrosis.

**TABLE 4:** ARCO staging at AVN diagnosis and radiological findings and symptoms with pain and activities of daily living at latest follow‐up.

Pt	Age at AVN diagnosis (years)	Hip joints	ARCO staging at diagnosis	Latest ARCO staging	Radiological outcome	Operative treatment	Interval from diagnostic to latest imaging (years)	Pain at latest follow‐up	ADL affected
1	18.4	Right	2A	2A	Static		12	No	No
2	11.4	Bilateral	Early 3A	Late 3A	Progression	Core decompression	2	No	No
3	14.3	Bilateral	3A	4	Progression		9.4	Yes	No
4	16.9	Right	2B	2B	Static		8.5	No	No
		Left	3C	4	Progression				
5	18.4	Right	2A	3B	Progression	Core decompression followed by bilateral arthroplasties	1.2	Yes	No
		Left	Early 3C	4	Progression				
6	18.9	Right	2A	2A	Static		6.3	No	No
		Left	2B	2B	Static				
7	17.5	Right	1C	4	Progression		5.6	Yes	Pain on prolonged walking
		Left	Early 3C	4	Progression				
8	15.1	Right	1C	1B	Improvement	Core decompression	3	No	No
		Left	1C	1C	Static				
9	18.2	Right	2C	4	Progression	Core decompression	5.3	Yes	No
		Left	1C	Early 3B	Progression				
10	15	Right	Early 3C	Late 3C	Progression		4	Yes	Need walking aid and sometimes wheelchair
		Left	Late 3A	Late 3A	Static				
11	17.6	Right	Late 1A	Early 1A	Improvement		2.3	No	No
12	11.3	Right	Early 3B	Late 2C	Progression	Core decompression	1.7	No	No
		Left	0	Late 3C	Progression				

Abbreviations: ADL, activities of daily living; ARCO, Association Research Circulation Osseous Classification; AVN, avascular necrosis; Pt, patient.

Five patients underwent orthopedic intervention with core decompression of femoral head with or without bone graft. Timing of intervention ranged from 1 months to 5 years after AVN diagnosis. One patient eventually required bilateral arthroplasties at 1 and 2 years for left and right hip, respectively.

In terms of symptom and functional outcomes, 7 out of 24 patients (29.2%) reported pain during follow‐up, five had hip involvement with two had previous surgical treatment for AVN. ARCO staging was grade 3 in three and grade 4 in five hip joints. Pain was mild in five patients and did not interfere with daily activities. These subjects did not require use of analgesics. Two had intermittent severe pain upon prolonged walking with one required the use of walking aids and wheelchair. For the seven patients with worsening of hip imaging, three patients with ARCO stage 3 and 4 changes did not report pain on follow‐up.

There is no statistically significant difference in survival outcome between AVN and non‐AVN groups with an overall survival of 100% versus 90.2% ± 1.4% (*p* = 0.099) and an event‐free survival of 91.5% ± 5.8% versus 82% ± 1.8% (*p* = 0.193), respectively.

### 3.6 Exploratory analysis

Among patients with AVN, more female patients tended to have progressive or worsening conditions than male patients (90% vs. 50%; *p*‐value 0.045). Severity of AVN and long‐term outcomes were not associated with other risk factors (type of treatment protocol, immunophenotype, NCI risk group, presenting white blood cell count, and cumulative dose of corticosteroid and asparaginase at the time of AVN diagnosis).

## 4 DISCUSSION

In this retrospective cohort study on AVN among Chinese children with ALL, the incidence of symptomatic AVN for patients ≥10 years old was 18.2%, which was comparable to Western studies and was higher than that reported in the CCCG‐ALL‐2015 study.^2^, ^5^, ^6^, ^9^, ^12^, ^13^, ^20^, ^27^, ^28^ Multiple joints involvement were common with hips and knees being the most commonly affected joints.^9^, ^22^ The incidence of symptomatic AVN in reported studies, including our cohort, might be underestimated as adolescent patients might not report the symptoms and some studies might not list AVN as reportable adverse events. Consistent with the existing literature, our analysis showed that older age of diagnosis was risk factor with age ≥10 years being the most important risk factor for development of AVN. The underlying mechanisms being age‐related factors including skeletal maturation, osseous blood vessel supply, dexamethasone clearance, changes in concentrations of coagulation factors, and hormonal changes that ultimately affecting bone morphology, metabolism, and nourishment.^5^ There was no significant difference between T‐ALL and B‐ALL. Regarding gender, the results were conflicting. Some studies found female sex as a risk factor,^19^, ^20^, ^21^, ^29^ while some did not find any association.^9^, ^13^, ^22^, ^30^ Our study supports the later statement.

Corticosteroids and asparaginase are extensively used in ALL treatments. The dosage and schedule of dexamethasone was showed to impact the development of AVN, while asparaginase was suggested to be associated with development of AVN via its negative effect on serum albumin level and clearance of dexamethasone.^11^, ^31^ In the CCG‐1961 study, it was found that alternate week dexamethasone scheduling significantly reduce incidence of AVN as compared with 21 days of continuous administration during reinduction.^7^, ^20^ In our cohort, the group of patients treated with ALL‐IC‐BFM 2002 protocol had continuous administration of dexamethasone in reinduction, whereas in subsequent CCLG‐ALL 2008 and CCCG‐ALL 2015 protocols, alternate week schedule was adopted. Despite the change in scheduling, we did not observe significant change in incidence of AVN. We postulate that the protective effect of alternate weeks dexamethasone scheduling was offset by an overall increase in steroid and asparaginase dosage across the three protocols from IC‐BFM 2002 to CCCG ALL 2015 (Table 1).^7^, ^11^, ^32^

Among Asians, there was limited data on this complication. A low incidence of AVN was reported in the CCCG‐ALL‐2015 study.^12^ The retrospective cohort study by the JACLS group also reported a low incidence, with a 5‐year cumulative incidence of 7.2% and 5.9% among adolescent patients in ALL‐97 and ALL‐02 studies, respectively.^18^ The low incidence was attributed to a lower cumulative dose of dexamethasone and no concomitant use of asparaginase with dexamethasone.^18^ Despite the total cumulative dose of corticosteroid was comparable in our ALL studies (3411–8206 mg/m^2^) and studies in Japan (6547–11,309 mg/m^2^), a higher dose of dexamethasone was administered across our three ALL trials. There was concomitant use of dexamethasone and asparaginase in our treatment protocols with much higher dose of asparaginase being used as compared with the JACLS protocols (80,000–468,000 IU/m^2^ vs. 60,000 IU/m^2^). All these factors could contribute to the higher risk of AVN in our cohort. Further studies are thus required to verify the potential modifiable treatment factors in reducing the development of AVN.

In our cohort, nearly 60% of hip joints showed progressive worsening on follow‐up while one‐third remained stable or showed some improvement on follow‐up. For other affected sites, a smaller proportion showed progression (42%), while a significant proportion remained static or improved without operation. Whether surgical treatment improve long term functional outcome cannot be concluded from our study.

There is no standard recommendation on screening asymptomatic subjects for early detection of AVN.^6^, ^22^ The study by Kawedia et al. identified AVN in 71.8% of subjects while only a quarter of them were symptomatic. Many asymptomatic subjects remained stable over time.^10^, ^11^ Kaste et al. reported early screening with MRI at 6.5–9 months from start of treatment identified extensive AVN of femoral head with significant risk of collapse, as defined by more than 30% articular surface involvement, in 5.6% of the subjects. Notably, 26 out of 30 patients identified were asymptomatic.^6^ Our study did not investigate on asymptomatic cases, but we demonstrated radiographic progressive disease occurred in many of our patients with symptomatic AVN and 25% required surgical intervention. It has been suggested that an earlier detection of AVN at an asymptomatic stage could mitigate progression and a better functional outcome.^33^

If screening is considered in ALL patients, it should focus on the group at increased risk of development of severe or progressive AVN. As supported by the findings in this and previous reported studies, patients ≥10 years was at the highest risk and should be considered as the target group for screening.^6^ In our cohort, the shortest duration from diagnosis of ALL to that of symptomatic AVN was 6 months and median time to AVN diagnosis was 1.2 years. This was consistent with the observations in previous report^6^ and supported the timing of screening to be after reinduction or during early maintenance phase of treatment.^33^

The limitations in this study included retrospective nature of the study and small sample size. The incidence could be underestimated as some patients might not report their symptoms especially in cases with mild pain. As most studies only report on symptomatic AVN without accounting those asymptomatic cases, we believe the underestimated incidence is comparable among these studies. Nevertheless, the incidence of AVN among our adolescent patients is much higher than that reported by CCCG and JACLS group and is comparable with Western studies. Our finding suggested that Chinese patients are also at considerable risk of developing this complication. Our study cohort is also limited by not having data on genetic variations which is now increasingly recognized to be related to the development of AVN in ALL individuals.^19^, ^34^, ^35^

## 5 CONCLUSION

The incidence of symptomatic AVN in Chinese children with ALL was comparable to reports in Western studies. Adolescent age ≥10 years old was recognized to be the most important factor for development of AVN. The overall clinical outcomes were satisfactory in majority of patients, though radiological progressive disease was commonly observed. Some patients required orthopedic intervention and a minority had persistent symptoms affecting functioning.

## AUTHOR CONTRIBUTIONS

**Calvin Pui Lun Hoo:** Conceptualization (lead); data curation (lead); formal analysis (lead); project administration (equal); writing – original draft (lead); writing – review and editing (lead). **Alex Wing Kwan Leung:** Conceptualization (lead); data curation (equal); formal analysis (lead); project administration (equal); supervision (lead); writing – original draft (lead); writing – review and editing (lead). **Joyce Pui Kwan Chan:** Data curation (lead); project administration (lead); writing – original draft (equal); writing – review and editing (equal). **Yin Ting Cheung:** Data curation (equal); formal analysis (equal); writing – original draft (equal); writing – review and editing (equal). **Frankie Wai Tsoi Cheng:** Writing – review and editing (equal). **Terry Tin Wai Chow:** Writing – review and editing (equal). **Grace Kee See Lam:** Writing – review and editing (equal). **Sau Yin Ha:** Writing – review and editing (equal). **Alan Kwok Shing Chiang:** Writing – review and editing (equal). **Rever Chak Ho Li:** Writing – review and editing (equal). **Chi Kong Li:** Conceptualization (equal); supervision (equal); writing – original draft (equal); writing – review and editing (equal).

## CONFLICT OF INTEREST STATEMENT

The authors declared that there is no potential conflicts of interest.

## Data Availability

The data that support the findings of this study are available from the corresponding author upon reasonable request.
